# The Physician’s Record of Disease and Engagements for 1856

**Published:** 1856-01

**Authors:** 


*The Physician's Record of Disease and Engagements for* 1856. Buffalo: Phinney & Co., Publishers.

When Lindsay & Blakiston issued their "Physicians' Visiting List," we thought it the perfection of convenience and compactness; as indeed it is so far as it goes. But this new Buffalo "Physician's Record" is equally convenient, and goes a great deal farther.

It is worth a careful description: First, you have a list of poisons and antidotes; second, a table of proportionate doses for different ages; and third, a space in which to record a medical and surgical feebill.

Then comes the "Record of Disease" which is *the* feature of the book. Two pages (facing each other) are ruled in fourteen columns. The first has space for thirty names of patients, and in succeeding columns the observer may register the date of attack, date of first medical visit, age, country, occupation, locality of residence, predisposing causes, exciting causes, name of disease, duration of disease, complicated with what, amount of medical attention, and character of termination. This department affords space for the registry of 1920 cases. May we have as many!

Following this we have the usual physician's visiting list.

This little pocket book, the preface tells us, was gotten up by the publishers at the request of the "Committee on Epidemics" for the Eighth District of the State of New York, which means, we suppose, that our friend, Dr. H. M. Conger, has been at the pains to perform the necessary labor, in order to secure full reports of disease in this district.

Our readers should recollect that all this matter is so compacted as to be easily tucked into a breast pocket, that it furnishes all the essentials of notes for transferring to the case-book in a uniform and excellent arrangement; and that, finally, it is a day book and journal in itself, with all the usual Dr. and Cr. conveniences, and they will then appreciate its merit.

Our friends at a distance may easily procure it by ordering from Messrs. Phinney & Co.

